# Regulation of HGF-induced hepatocyte proliferation by the small GTPase Arf6 through the PIP_2_-producing enzyme PIP5K1A

**DOI:** 10.1038/s41598-017-09633-z

**Published:** 2017-08-25

**Authors:** Meng-Tsz Tsai, Naohiro Katagiri, Norihiko Ohbayashi, Kenichi Iwasaki, Nobuhiro Ohkohchi, Shih-Torng Ding, Yasunori Kanaho, Yuji Funakoshi

**Affiliations:** 1 Department of Physiological Chemistry, University of Tsukuba, 1-1-1 Tennodai, Tsukuba, 305-8575 Japan; 2 Department of Gastrointestinal and Hepato-Biliary-Pancreatic Surgery, Faculty of Medicine and Graduate School of Comprehensive Human Science, University of Tsukuba, 1-1-1 Tennodai, Tsukuba, 305-8575 Japan; 3 Depatment of Animal Science and Technology, National Taiwan University, No. 50, Ln. 155, Sec. 3, Keelung Rd., Da’an Dist., Taipei City, 106 Taiwan

## Abstract

HGF and its receptor c-Met are critical molecules in various biological processes. Others and we have previously shown that the small GTPase Arf6 plays a pivotal role in HGF signaling in hepatocytes. However, the molecular mechanism of how Arf6 regulates HGF signaling is unclear. Here, we show that Arf6 plays an important role in HGF-stimulated hepatocyte proliferation and liver regeneration through the phosphatidylinositol 4,5-bisphosphate (PIP_2_)-producing enzyme PIP5K1A. We find that knockdown of Arf6 and PIP5K1A in HepG2 cells inhibits HGF-stimulated proliferation, Akt activation, and generation of phosphatidylinositol 3,4,5-trisphosphate (PIP_3_) and its precursor PIP_2_. Interestingly, PIP5K1A is recruited to c-Met upon HGF stimulation in an Arf6 activity-dependent manner. Finally, we show that hepatocyte proliferation and liver regeneration after partial hepatectomy are suppressed in *Pip5k1a* knockout mice. These results provide insight into the molecular mechanism for HGF-stimulated hepatocyte proliferation and liver regeneration: Arf6 recruits PIP5K1A to c-Met and activates it upon HGF stimulation to produce PIP_2_ and subsequently PIP_3_, which in turn activates Akt to promote hepatocyte proliferation, thereby accelerating liver regeneration after liver injury.

## Introduction

Hepatocyte proliferation is tightly regulated by cellular signaling pathways stimulated by various growth factors and cytokines^1^, and fundamental for fetal liver development and regeneration induced after physical, infectious and toxic injury of the liver. Hepatocyte growth factor (HGF), which was originally discovered in the serum and platelets of rats^2–4^, is a potent mitogen regulating cell proliferation, morphogenesis and motility through activation of the tyrosine kinase receptor c-Met^5^. It has been shown that the HGF/c-Met axis plays crucial roles in adult liver regeneration, embryonic organ development and wound healing^6^ through phosphoinositide 3-kinase (PI3K)/Akt and Ras/Raf/extracellular signal-regulated kinase (Erk) cascade pathways^7^.

In addition to the downstream signaling cascade of HGF/c-Met axis described above, others and we have reported that the small GTPase ADP-ribosylation factor 6 (Arf6) is involved in an HGF-stimulated signaling pathway(s) to regulate biological processes^8–11^. Arf6 localizes at the plasma membrane and endosomal compartments, and functions as the molecular switch in the cellular signaling pathways by cycling between GDP-bound inactive and GTP-bound active forms. This cycle is precisely regulated by guanine nucleotide exchange factors (GEFs) and GTPase-activating proteins (GAPs): Arf6 GEFs promote the exchange of GDP for GTP to activate Arf6 in response to agonist stimulation of the cell, and Arf6 GAPs stimulate the GTPase activity of Arf6 to accelerate hydrolysis of Arf6-bound GTP to GDP to inactivate Arf6. Activated Arf6 regulates location and activity of downstream effectors and eventually various cellular events such as phosphoinositide metabolism, membrane trafficking and actin cytoskeleton reorganization^12^.

We have previously identified type I phosphatidylinositol 4-phosphate 5-kinase (PIP5K1) as a downstream effector of Arf6^13^. In mammals, three PIP5K1 isozymes, PIP5K1A (corresponding to human PIP5Kα or mouse PIP5Kβ), PIP5K1B (corresponding to human PIP5Kβ or mouse PIP5Kα) and PIP5K1C (corresponding to human and mouse PIP5Kγ), have been identified. They catalyze phosphorylation of phosphatidylinositol 4-phosphate to generate the pleiotropic lipid messenger phosphatidylinositol 4,5-bisphosphate [PI(4,5)P_2_ or PIP_2_] that regulates localization and activity of various target proteins^14^. PIP_2_ also serves as a precursor of lipid second messengers: It is hydrolyzed by phospholipase C to generate two lipid messengers, diacylglycerol and inositol 1,4,5-triphosphate, or further phosphorylated by phosphoinositide 3-kinase (PI3K) to yield phosphatidylinositol 3,4,5-trisphosphate [PI(3,4,5)P_3_ or PIP_3_], which activates the protein kinase Akt, thereby regulating cell proliferation and survival^15, 16^. Thus, Arf6 regulates various cellular functions through the activation of its downstream effector PIP5K1.

In the previous study, we have reported that *Arf6* knockout mice are embryonic lethal with a severe defect in the liver development^10^. We have also shown that HGF-dependent *in vitro* cord formation by primary cultured hepatocytes is significantly impaired in *Arf6*-deleted hepatocytes^10^. These observations strongly suggest that Arf6 plays a crucial role in the HGF signaling pathway in hepatocytes. However, the molecular mechanism of how Arf6 is involved in the HGF signaling in hepatocytes has not yet been fully understood. Here, we find a novel molecular mechanism for HGF-dependent hepatocyte proliferation, in which Arf6 and its downstream effector PIP5K1A mediate HGF signaling. We also show that liver regeneration after liver injury is impaired in *Pip5k1a* knockout mice, providing a novel insight into the molecular mechanism of the liver regeneration.

## Results

### Arf6 is essential for Akt activation to promote HGF-dependent proliferation of HepG2 cells

**Figure 1:**
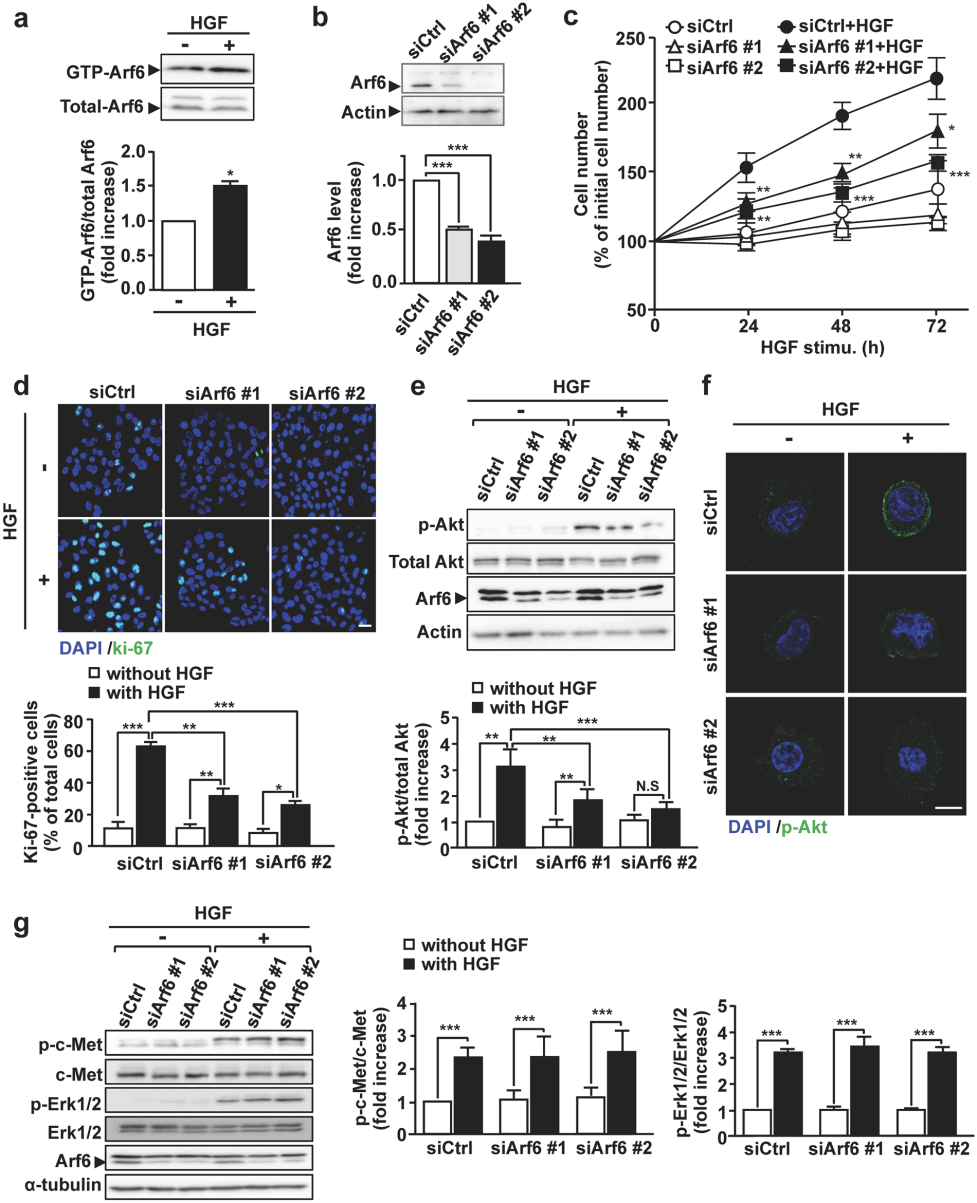
Arf6 is essential for Akt activation to promote HGF-dependent proliferation of HepG2 cells. (**a**) After HepG2 cells were stimulated without or with 10 ng/mL of HGF at 37 °C for 10 min, active GTP-Arf6 in the cell was analyzed by Western blotting with anti-Arf6 antibody (*upper panels*) and quantified (*lower panel*). (**b**) HepG2 cells were transfected with 10 nM of control siRNA (siCtrl) or siRNAs for Arf6 (siArf6 #1 and siArf6 #2). After 48 h of transfection, Arf6 protein levels were detected by Western blotting (*upper panels*) and quantified (*lower panel*). (**c**) HepG2 cells transfected with 10 nM of siRNAs for control and Arf6 were stimulated without or with 10 ng/mL of HGF at 37 °C for the indicated time, and the cell number was counted. (**d**) HepG2 cells transfected with siRNAs for control and Arf6 were stimulated with HGF for 24 h and stained for the proliferation marker Ki-67 (green) and for nuclei with DAPI (blue) (*upper panels*), and Ki-67-positive cells were counted (*lower panel*). Scale bar, 20 μm. (**e**) HepG2 cells transfected with siRNAs for control and Arf6 were stimulated with HGF as in Fig. 1a. Proteins in cell lysates were immunoblotted with antibodies for the indicated proteins (*upper panels*), and p-Akt levels were quantified (*lower panel*). (**f**) HepG2 cells transfected with siRNAs for control and Arf6 were stimulated with HGF as in Fig. 1a, and immunostained for p-Akt (green) and for nuclei with DAPI (blue). Scale bar, 10 μm. (**g**) HepG2 cells transfected with siRNAs for control and Arf6 were stimulated with HGF as in Fig. 1a. Proteins in cell lysates were immunoblotted with antibodies for the indicated proteins (*left panels*), and levels of p-c-Met and p-Erk were quantified (*middle and right panels*, respectively). All quantitative data represent means ± SEM from at least three independent experiments. Statistical analyses: Student *t*-test (**a**), one-way ANOVA with post hoc Tukey’s test (**b**), and two-way ANOVA with post hoc Bonferroni’s test (**c**,**d**,**e** and **g**). *p < 0.05; **p < 0.01; ***p < 0.001. Blots shown in (**a**,**b**,**e** and **g**) are cropped images. The full-length blots are presented in Supplementary Figure.

To investigate the role of Arf6 in HGF-dependent cell functions of hepatocytes, we employed the human hepatocellular carcinoma cell line HepG2 cells as a model system. As we have previously demonstrated with fetal mouse hepatocytes^10^, Arf6 in HepG2 cells was also activated in response to HGF stimulation (Fig. 1a). Knockdown of Arf6 in these cells attenuated HGF-stimulated cell proliferation as assessed by counting cell number and immunostaining the proliferation marker Ki-67 (Fig. 1b–d). These results demonstrate that Arf6 mediates HGF signaling to regulate cell proliferation in HepG2 cells.

The PI3K/Akt axis is a key pathway of HGF-dependent cell proliferation^15^. Since Akt is recruited to the plasma membrane and phosphorylated by phosphoinositide-dependent kinase 1 to be activated, we examined the involvement of Arf6 in this process. HGF-stimulated Akt phosphorylation and increase in the phosphorylated-Akt (p-Akt) level at the plasma membrane were significantly suppressed by knockdown of Arf6 (Fig. 1e,f). On the other hand, HGF-dependent phosphorylation of Erk was not affected by knockdown of Arf6 (Fig. 1g), ruling out the involvement of Arf6 in the Ras/Raf/Erk pathway, another key pathway downstream of c-Met. The inhibition of Akt phosphorylation is unlikely to be attributable to the suppression of c-Met: the HGF-stimulated phosphorylation level of c-Met was not affected by Arf6 knockdown (Fig. 1g). These results, taken together, demonstrate that Arf6 regulates the HGF-dependent Akt recruitment to the plasma membrane and its subsequent activation to promote hepatocyte proliferation.

### Arf6 promotes PIP_2_ and PIP_3_ generation by activating PIP5K1A upon HGF stimulation

**Figure 2:**
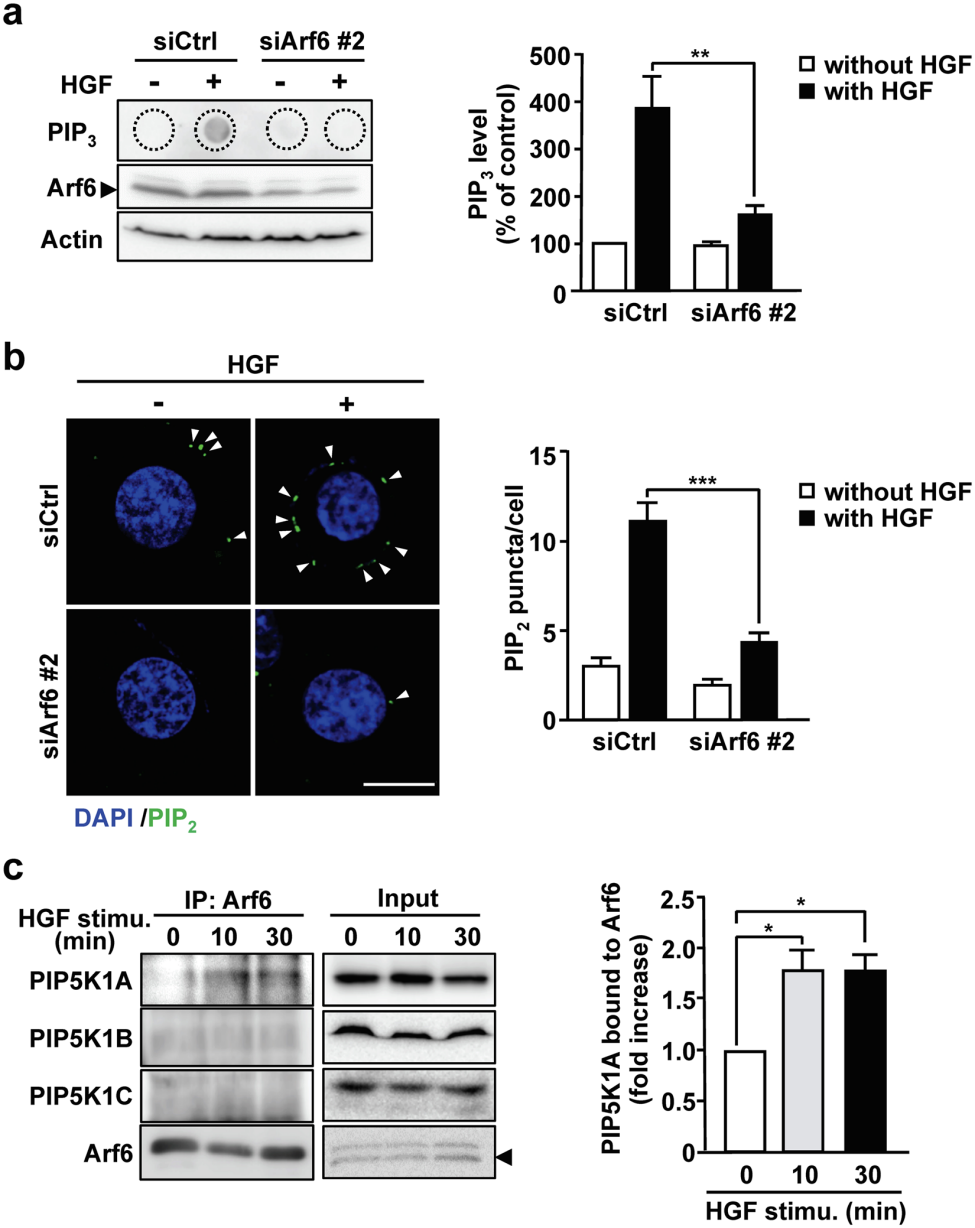
Arf6 promotes PIP_2_ and PIP_3_ production by activating PIP5K1A upon HGF stimulation of HepG2 cells. (**a**) HepG2 cells transfected with siRNAs for control and Arf6 were stimulated with HGF as in Fig. 1a. After lipids were extracted from the cells, PIP_3_ was detected by dot blotting with anti-PIP_3_ antibody (*left panels*). Arf6 and actin in cell lysates were also detected by Western blotting. PIP_3_ levels were quantified (*right panel*). (**b**) HepG2 cells transfected with siRNAs for control and Arf6 were stimulated with HGF as in Fig. 1a, and immunostained for PIP_2_ (green) and stained for nuclei with DAPI (blue) (*left panels*). The number of PIP_2_ puncta shown by arrow heads in the images was counted and normalized by cell number (*right panel*). Scale bar, 10 μm. (**c**) HepG2 cells were stimulated with 10 ng/mL of HGF for the indicated time. Endogenous Arf6 was immunoprecipitated, and co-precipitated PIP5K1 isoforms were detected by Western blotting with antibodies specific to each PIP5K1 isoform (*left panels*), and co-precipitated PIP5K1A was quantified (*right panel*). All quantitative results represent means ± SEM from at least three independent experiments. Statistical analyses: two-way ANOVA with post hoc Bonferroni’s test (**a** and **b**) and one-way ANOVA with post hoc Tukey’s test (**c**). *p < 0.05; **p < 0.01; ***p < 0.001. Blots shown in (**a** and **c**) are cropped images. The full-length blots are presented in Supplementary Figure.

Since the recruitment of Akt to the plasma membrane is mediated by PIP_3_ generated in response to various agonists including HGF^17^, we examined whether Arf6 is involved in the HGF-dependent PIP_3_ generation. HGF stimulation of HepG2 cells markedly increased the PIP_3_ production, which was almost completely suppressed by knockdown of Arf6 (Fig. 2a), suggesting that Arf6 is involved in HGF-dependent PIP_3_ production.

PIP_3_ is generated by phosphorylation of PIP_2_ by PI3K. We have previously demonstrated that Arf6 directly activates the PIP_2_-generating enzyme PIP5K1^13^. These observations led us to speculate that Arf6 activates PIP5K1 to generate the PI3K substrate PIP_2_ upon HGF stimulation, thereby contributing to the increase in the PIP_3_ levels. To address this assumption, effects of Arf6 knockdown on the PIP_2_ level were analyzed by immunocytochemistry with the PIP_2_-specific antibody. As was expected, HGF stimulation of HepG2 cells increased the PIP_2_ level at the plasma membrane, and knockdown of Arf6 significantly suppressed the production of PIP_2_ (Fig. 2b). Furthermore, it was found that PIP5K1A, but not PIP5K1B and PIP5K1C, interacted with Arf6 upon HGF stimulation of HepG2 cells (Fig. 2c). These results, taken together with the result shown in Fig. 1a, suggest that Arf6 activated by HGF stimulation interacts with and positively regulates PIP5K1A to produce the PI3K substrate PIP_2_ at the plasma membrane, thereby increasing the PIP_3_ level.

### Arf6 activated by HGF stimulation forms a complex with PIP5K1A and c-Met

**Figure 3:**
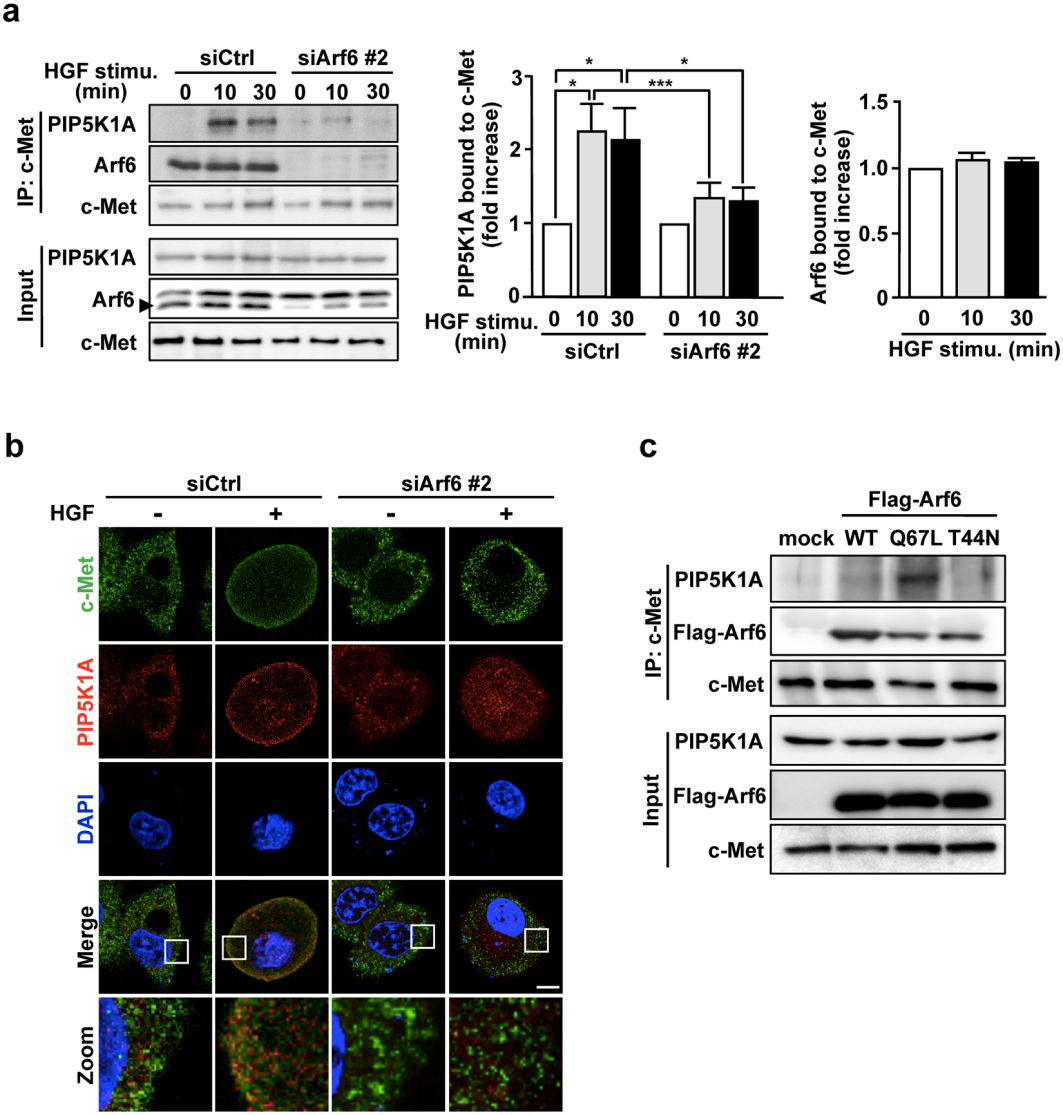
Arf6 activated by HGF stimulation forms a complex with PIP5K1A and c-Met. (**a**) After HepG2 cells transfected with siRNAs for control and Arf6 were stimulated with 10 ng/mL of HGF for the indicated time, c-Met was immunoprecipitated with anti-c-Met antibody, and co-precipitated Arf6 and PIP5K1A were assessed by Western blotting with anti-Arf6 and anti-PIP5K1A antibodies, respectively (*left panels*). Signal intensities of co-precipitated PIP5K1A (*middle panel*) and Arf6 (*right panel*) were quantified. Shown are means ± SEM from three independent experiments. Statistical analyses: two-way ANOVA with post hoc Bonferroni’s test (for PIP5K1A bound to c-Met) and one-way ANOVA with post hoc Tukey’s test (for Arf6 bound to c-Met). *p < 0.05; ***p < 0.001. (**b**) HepG2 cells transfected with siRNAs for control and Arf6 were stimulated with HGF as in Fig. 1a, and immunostained for c-Met and PIP5K1A. Nuclei were also stained with DAPI. Area of squares in images were magnified and shown in the bottom. Scale bar, 10 μm. Green, c-Met; Red, PIP5K1A; Blue, DAPI. (**c**) HepG2 cells were transfected with a control plasmid and plasmids for Flag-tagged Arf6 mutants, Q67L and T44N. After c-Met was immunoprecipitated, co-precipitated PIP5K1A and Flag-Arf6 mutants were assessed by Western blotting with anti-PIP5K1A and -Flag antibodies, respectively. Blots shown in (**a** and **c**) are cropped images. The full-length blots are presented in Supplementary Figure.

The results obtained above, taken together with the report that Arf6 recruits its effector protein GGA3 (Golgi-localized, gamma adaptin ear-containing, Arf-binding 3) to c-Met^18^, raised a possibility that activated Arf6 forms a complex with PIP5K1A and c-Met. To address this issue, interaction of c-Met with PIP5K1A and Arf6 was assessed by immunoprecipitation assay. As expected, interaction between c-Met and PIP5K1A was markedly enhanced by HGF stimulation, and knockdown of Arf6 drastically inhibited this interaction (Fig. 3a). Interestingly, under these conditions, Arf6 constitutively interacted with c-Met (Fig. 3a). Consistent with the results obtained by immunoprecipitation assay, PIP5K1A predominantly locating in the cytosol in the resting state of the cell translocated to the plasma membrane and colocalized with c-Met upon HGF stimulation, which was again attenuated by knockdown of Arf6 (Fig. 3b).

As Arf6 was activated by HGF stimulation as shown in Fig. 1a, the results shown above indicate that Arf6 activated by HGF stimulation is responsible for the PIP5K1A recruitment to the plasma membrane to form a complex with c-Met. To test this assumption, Q67L and T44N mutants of Arf6, which mimic GTP-bound active and GDP-bound inactive forms of Arf6, respectively, were expressed in HepG2 cells and their effects on the interaction of PIP5K1A with c-Met were analyzed. The interaction was observed in the cell overexpressed with Q67L but not with T44N (Fig. 3c), supporting the notion that the active form of Arf6 recruits PIP5K1A to c-Met. Consistent with the result shown in Fig. 3a, Q67L and T44N mutants were both found to interact with c-Met (Fig. 3c).

### PIP5K1A is required for HGF-dependent Akt activation and subsequent proliferation of hepatocytes

**Figure 4:**
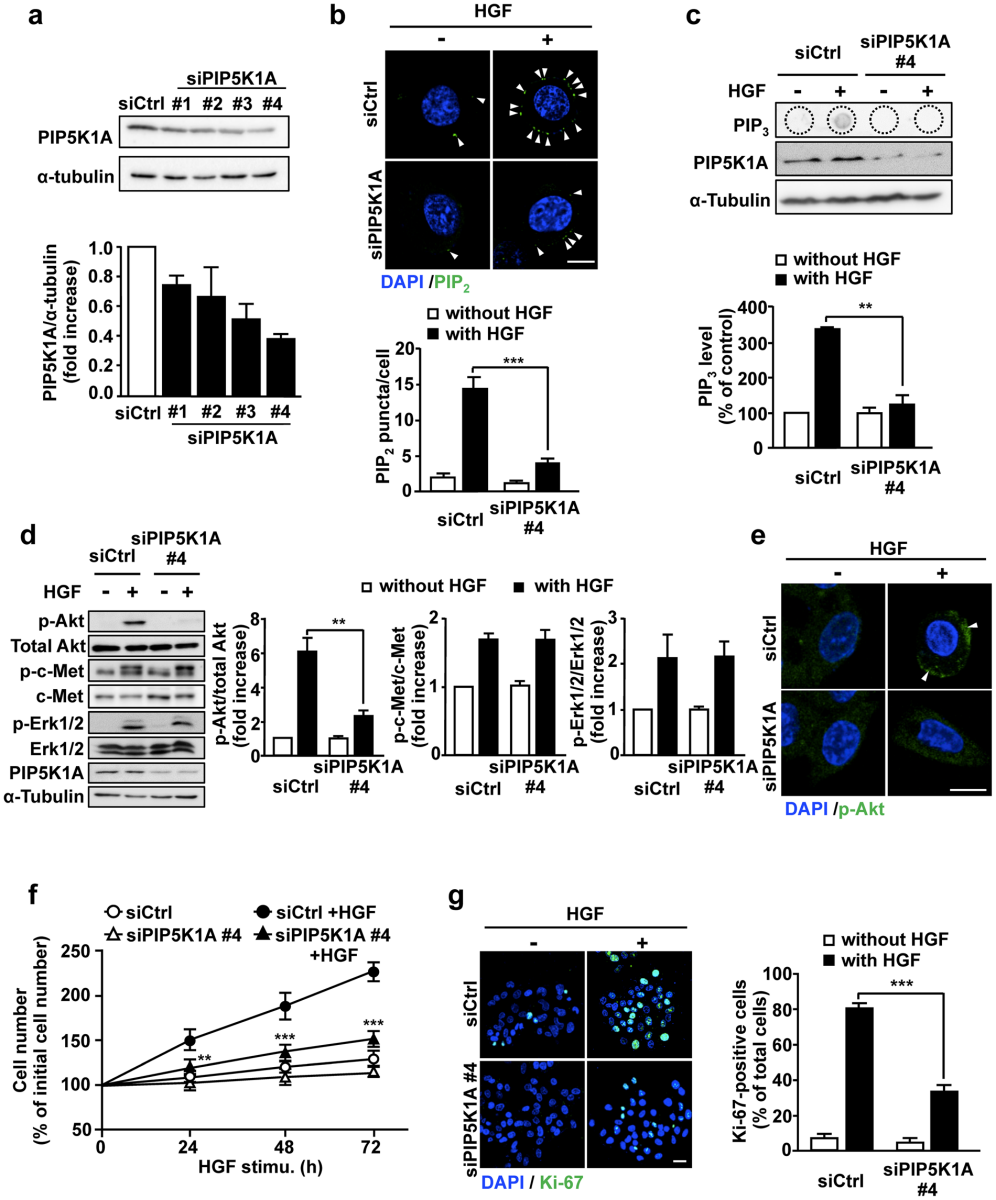
Knockdown of PIP5K1A in HepG2 cells suppresses HGF-dependent PIP_2_ and PIP_3_ production, Akt activation and cell proliferation. (**a**) HepG2 cells were transfected with 10 nM of siRNAs for control and for PIP5K1A, and the PIP5K1A protein levels were detected by Western blotting (*upper panels*) and quantified (*lower panel*). (**b–e**) HepG2 cells transfected with siRNAs for control and PIP5K1A were simulated with HGF as in Fig. 1a, and PIP_2_ production (**b**), PIP_3_ production (**c**), Akt phosphorylation (**d**), and subcellular localization of p-Akt (**e**) were assessed as in Figs 2b, 2a, 1e and 1f, respectively. Phosphorylation of c-Met and Erk (**d**) were assessed as in Fig. 1g. Nuclei were also stained with DAPI (**b**,**e**). Green, PIP_2_ in (**b**) and p-Akt in (**e**); Blue, DAPI. Scale bars, 10 μm. (**f**,**g**) HGF-dependent proliferation of PIP5K1A-knocked-down HepG2 cells was assessed by counting cell number (**f**) and staining Ki-67 (**g**). Scale bar, 20 μm. Nuclei were also stained with DAPI in Fig. 4g. The quantitative results shown are means ± SEM from at least three independent experiments. Statistical analyses: two-way ANOVA with post hoc Bonferroni’s test. **p < 0.01; ***p < 0.001. Blots shown in (**a**,**c**) and (**d**) are cropped images. The full-length blots are presented in Supplementary Figure.

To confirm the notion that PIP5K1A is involved in the PIP_2_ and PIP_3_ generation, Akt phosphorylation and cell proliferation through the production of the PI3K substrate PIP_2_ upon HGF stimulation, we employed siRNAs that efficiently knocked down PIP5K1A in HepG2 cells (Fig. 4a). Knockdown of PIP5K1A significantly suppressed the HGF-dependent PIP_2_ and PIP_3_ production (Fig. 4b,c), suggesting that PIP5K1A is the major enzyme to provide the PI3K substrate PIP_2_. Consistent with our notion, Akt phosphorylation and the accumulation of p-Akt at the plasma membrane induced by HGF stimulation were significantly suppressed in PIP5K1A-knocked-down cells, while phosphorylation of c-Met and Erk were not affected (Fig. 4d,e). Finally, HGF-dependent proliferation of HepG2 cells, which was assessed by cell number (Fig. 4f) and Ki-67 staining (Fig. 4g), was also inhibited by PIP5K1A knockdown. Thus, these results strongly support our notion described above.

**Figure 5:**
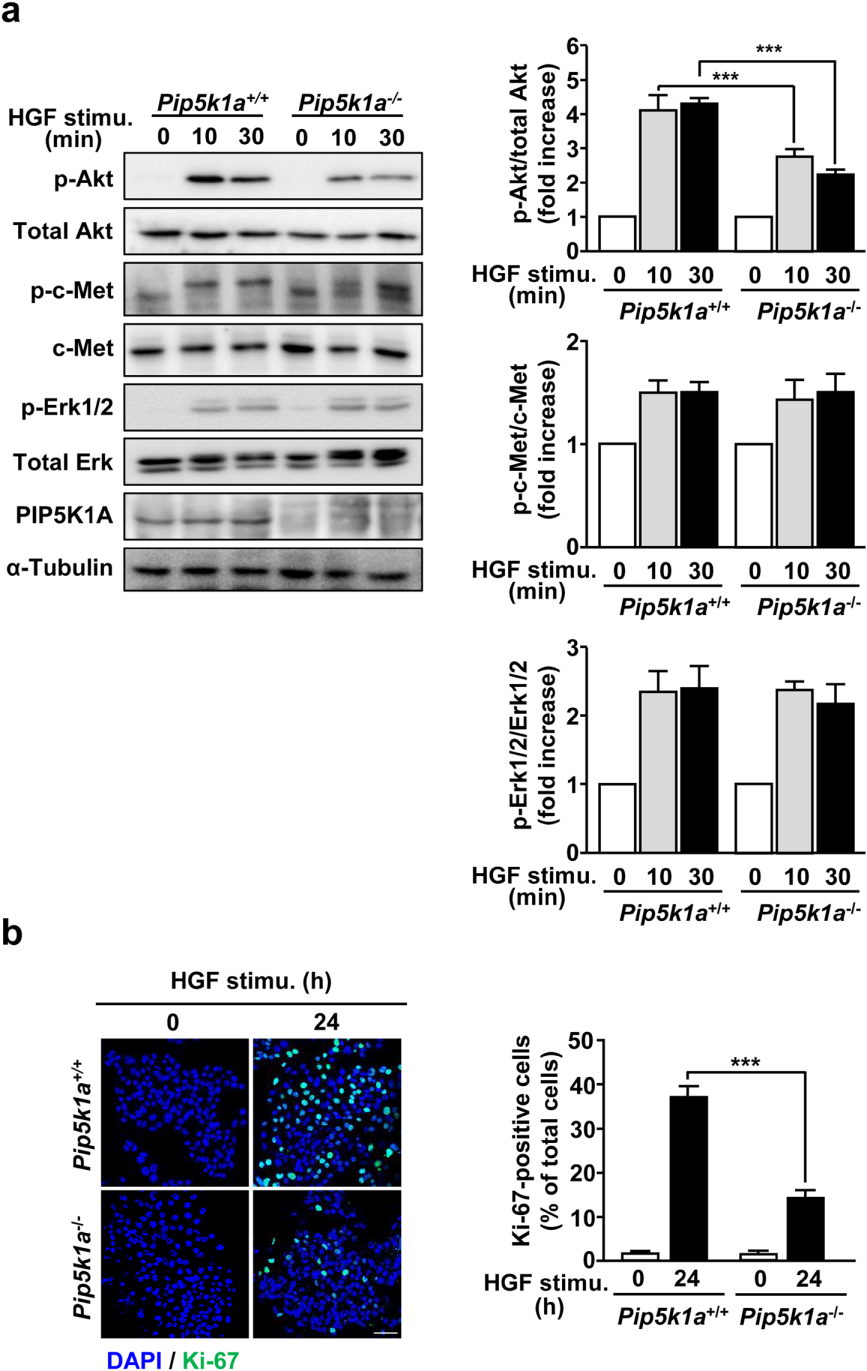
HGF-promoted Akt phosphorylation and cell proliferation are impaired in *Pip5K1a* ^−*/−*^ hepatocytes. Primary hepatocytes prepared from adult *Pip5k1a* ^*+/+*^ (control) and *Pip5k1a* ^−*/−*^ mice were stimulated with 10 ng/mL of HGF for the indicated time, and Akt phosphorylation (**a**) and cell proliferation (**b**) were assessed as in Fig. 1e and d, respectively. Phosphorylation of c-Met and Erk (**a**) were also assessed as in Fig. 1g. Quantitative results represent means ± SEM from at least three independent experiments. Scale bar, 50 μm. Statistical analyses: two-way ANOVA with post hoc Bonferroni’s test. ***p < 0.001. Blots shown in (**a**) are cropped images. The full-length blots are presented in Supplementary Figure.

To investigate whether the results obtained above with HepG2 cells are also the case in hepatocytes, we isolated hepatocytes from adult *Pip5k1a* ^−*/−*^ mice and analyzed Akt phosphorylation and proliferation stimulated by HGF. Consistent with the results obtained with HepG2 cells, these HGF-dependent phenomena were impaired in *Pip5k1a* ^−*/−*^ primary hepatocytes, whereas levels of p-c-Met and p-Erk1/2 were comparable to those of control cells (Fig. 5a,b). These results provide evidence that PIP5K1A plays an important role in HGF-dependent hepatocyte proliferation through the activation of Akt.

### PIP5K1A is involved in liver regeneration after partial hepatectomy

**Figure 6:**
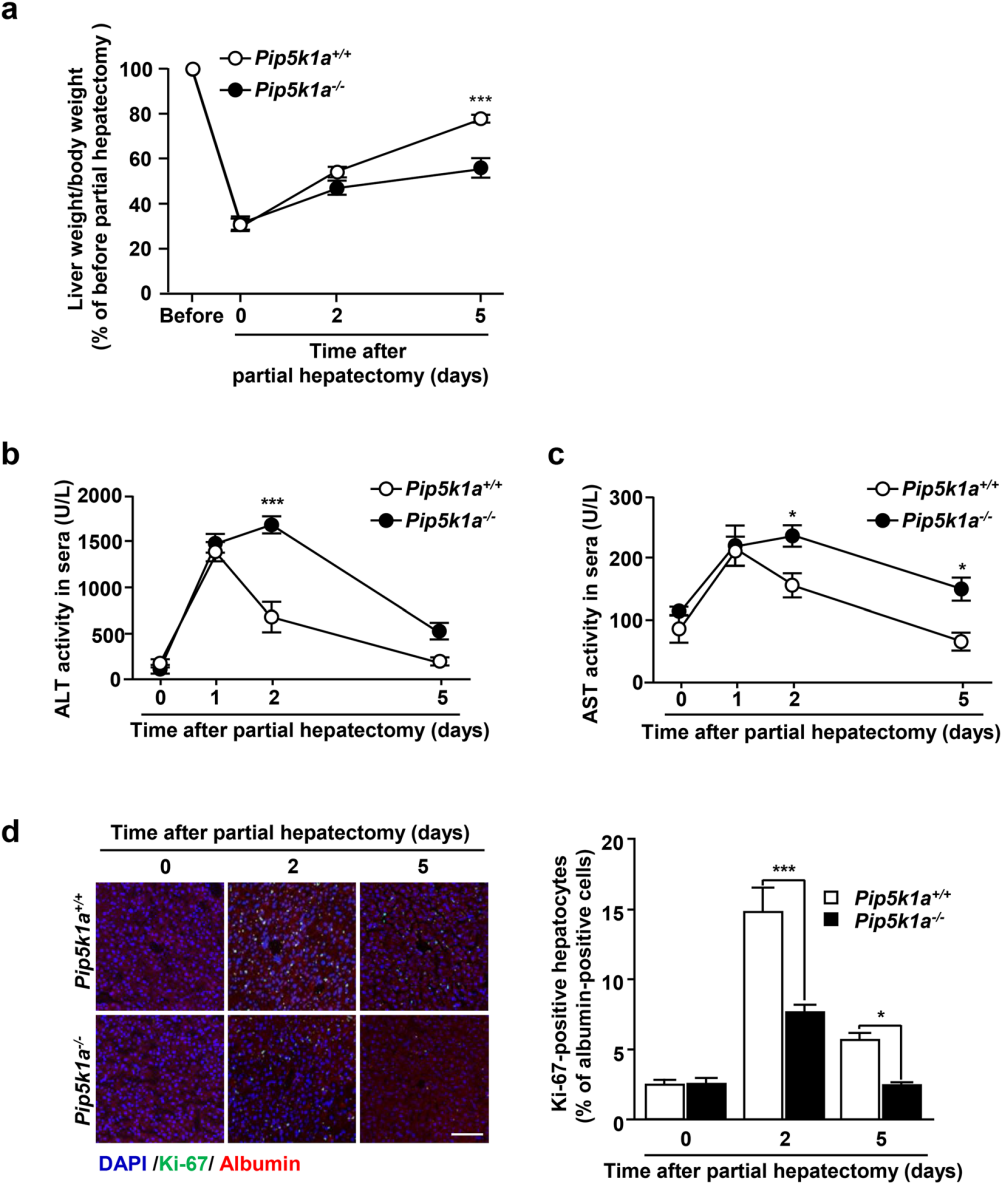
Deletion of *Pip5k1a* impairs liver regeneration after partial hepatectomy. (**a**) Livers of *Pip5k1a* ^*+/+*^ and *Pip5k1a* ^−*/−*^ mice were resected by 70% as described in *Materials and Methods*. At the indicated time after partial hepatectomy, livers were excised from mice, and ratios of liver weight/body weight were calculated. (**b,c**) Sera were collected from the mice in (**a**) at the indicated time after partial hepatectomy, and ALT (**b**) and AST (**c**) activities in sera were measured. (**d**) Frozen liver sections prepared from control and *Pip5k1a* ^−*/−*^ mice at the indicated time after partial hepatectomy were subjected to immunostaining for Ki-67 (green) and albumin (red) (*left panels*). Nuclei were also stained with DAPI (blue). Scale bar, 100 μm. The number of Ki-67-positive hepatocytes was counted (*right panel*). All quantitative results represent means ± SEM (n = 4 for each genotype). Statistical analyses: two-way ANOVA with post hoc Bonferroni’s test. *p < 0.05; ***p < 0.001.

*Arf6* ^−*/−*^ mice exhibit severe defect in liver development^10^, while *Pip5k1a* ^−*/−*^ mice did not show obvious defects in embryonic liver development^19^. Nevertheless, the results obtained above demonstrate that PIP5K1A functions as a downstream effector of Arf6 in the HGF-dependent cellular signaling pathway regulating hepatocyte proliferation. This conclusion and the fact that HGF plays a pivotal role in liver regeneration^5, 20^, which absolutely requires hepatocyte proliferation, led us to speculate that PIP5K1A is involved in liver regeneration after liver injuries, but not in embryonic liver development. To address this issue, partial hepatectomy was employed to examine the significance of PIP5K1A in liver regeneration. In control mice, the liver weight recovered to 77.7 ± 1.57% of the non-resected liver weight after 5 days of 70% hepatectomy, while the recovery was slower in *Pip5k1a* ^−*/−*^ mice (Fig. 6a): the liver weight at 5 days after hepatectomy was 55.6 ± 3.86% of the non-resected liver weight. Analyses of the levels of alanine transaminase (ALT) and aspartate aminotransferase (AST), the liver injury markers released from hepatocytes into the serum, also revealed that repair of the injured liver was slower in *Pip5k1a* ^−*/−*^ mice (Fig. 6b,c): AST and ALT activities in sera of control mice were elevated at 1 day after partial hepatectomy and gradually decreased, reaching the basal level after 5 days, while they were significantly higher at 2 days and higher levels sustained till 5 days in *Pip5k1a* ^−*/−*^ mice. Finally, the increase in the number of proliferating hepatocytes observed after partial hepatectomy was significantly suppressed in *Pip5k1a* ^−*/−*^ mice (Fig. 6d). However, we cannot totally rule out a possibility that PIP5K1A in other tissues is also contributing to liver regeneration, since PIP5K1A in whole body was knocked out in *Pip5k1a* ^−*/−*^ mice. Nevertheless, these results demonstrate that PIP5K1A is required for liver regeneration after partial hepatectomy and hepatocyte proliferation during this event.

## Discussion

**Figure 7:**
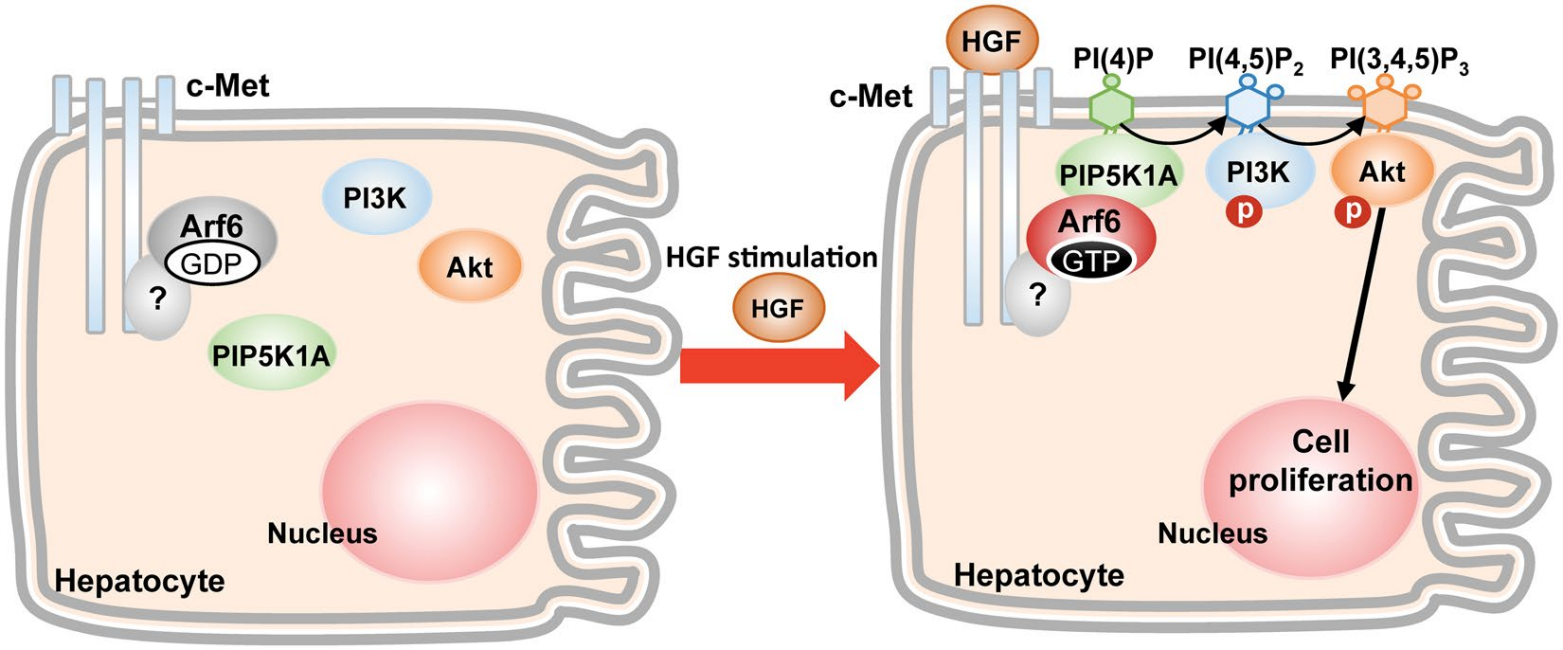
A model for molecular mechanism of HGF-induced hepatocyte proliferation. In this model, it is not clarified whether Arf6 directly interacts with c-Met or an unidentified factor mediates the interaction between Arf6 and c-Met.

In the present study, we provide evidence that Arf6 directly or indirectly bound to c-Met is activated by HGF stimulation of hepatocytes, and activated Arf6 recruits PIP5K1A to c-Met and activates it to produce the PI3K substrate PIP_2_, which is converted to PIP_3_ to activate Akt, thereby stimulating the HGF-dependent hepatocyte proliferation (Fig. 7). This model for the HGF signaling pathway mediated by Arf6 and PIP5K1A in hepatocyte proliferation gives insight into the molecular mechanism for liver regeneration after the liver injury.

In this HGF signaling pathway, we speculated that Arf6, which constitutively interacts with c-Met, recruits PIP5K1A to c-Met, when it is activated by HGF stimulation. Although the active form of Arf6 can directly activate PIP5K1^13^, we cannot totally exclude a possibility that another protein(s), which functions as a downstream effector of c-Met, facilitates or mediates the PIP5K1A recruitment to c-Met. This possibility is supported by the report that Arf6 and the c-Met adaptor protein Crk, which is involved in c-Met signaling by interacting with multiple downstream signaling molecules^18^, cooperate to recruit the Arf6 effector protein GGA3 to c-Met. Thus, an unidentified adaptor protein of c-Met may be a key determinant to recruit PIP5K1A to HGF-stimulated c-Met. Arf6 is capable of activating all PIP5K1 isozymes *in vitro* ^13^. If our assumption is true and there exist several adaptor proteins of c-Met, each of which specifically interacts and recruits each of three PIP5K1 isozymes to cellular compartments in cooperation with Arf6, adaptor proteins determine PIP5K1 isozymes to be spatiotemporally activated by Arf6 in an agonist type-dependent manner. It is of interest to identify whether there exist adaptor proteins that specifically interact with PIP5K1 isozymes to regulate their translocation dependently upon the types of agonists.

It has been suggested that the phosphorylation of PIP5K1A suppresses its lipid kinase activity^21, 22^. Based on their reports, it is plausible that c-Met activation dephosphorylates PIP5K1A by activating a phosphatase(s) and/or inhibiting a kinase(s) to stimulate its kinase activity cooperatively with Arf6. Alternatively, phosphorylation of PIP5K1A affects binding to and activation by Arf6. It is of interest to investigate these points.

Another question raised in this study is how Arf6 is activated by HGF stimulation. Arf6 activation upon agonist stimulation is precisely regulated by Arf6-specific GEFs, which promote the exchange of GDP on Arf6 for GTP. We have recently suggested that Grp1, EFA6B and EFA6D, of 7 Arf6-specific GEFs so far identified in mammalian cells, spatiotemporally activate Arf6 in the HGF-stimulated vascular endothelial cell to regulate β1 integrin recycling that is the critical cellular event for the HGF-induced tumor angiogenesis^11^. Attar *et al*. have shown that ARNO activates Arf6 in HGF-stimulated epithelial cells to regulate cell migration^23^. In addition, inhibition of cytohesins such as ARNO and Grp1 by the specific cytohesin inhibitor SecinH3 revealed that these cytohesin family members are required for the Arf6 activation in the signaling pathway for the HGF-mediated renal recovery after acute kidney injury^24^. Thus, several Arf6-sepcific GEFs are the possible candidates for the Arf6 activation in the signaling pathway of HGF-stimulated hepatocyte proliferation. Further investigation, *e.g*., effects of knockdown of Arf6-specific GEFs on HGF-stimulated hepatocyte proliferation and analysis of their subcellular localization by immunocytochemistry, would identify an Arf6-specific GEF involved in the activation of Arf6 in response to the HGF stimulation of hepatocytes.

HGF-stimulated hepatocyte proliferation through the Arf6-PIP5K1A axis is strongly suggested to be a crucial cell event for liver regeneration (Fig. 6). Although *Arf6* ^−*/−*^ mice exhibit embryonic lethality with a severe defect in liver development^10^, *Pip5k1a* ^−*/−*^ mice did not show obvious histological abnormality in the fetal liver up to 12 months^19^. These observations, taken together with the results obtained in this study, suggest that the Arf6-PIP5K1A axis is an important signaling pathway downstream of HGF/c-Met for liver regeneration in adult mice, but not for the fetal liver development: Arf6 activated by HGF stimulation of hepatocytes or hepatoblasts utilizes another unidentified downstream molecule(s) in the fetal liver development. This idea is supported by the observations obtained in this study that before the liver injury by partial hepatectomy, weight, histological structure and proliferating cells of the liver in *Pip5k1a* ^−*/−*^ mice did not show obvious differences from those of control mice (Fig. 6a,d). Thus, it is plausible that hepatocyte proliferation regulated by the Arf6-PIP5K1A axis downstream of HGF/c-Met might be a limited and crucial event for regeneration after liver damages, but not for fetal liver development.

We have previously demonstrated that fetal hepatocytes isolated from *Arf6* ^−*/−*^ embryos exhibit no defect in HGF-stimulated proliferation^10^, while knockdown of Arf6 or its downstream effector PIP5K1A and deletion of PIP5K1A significantly suppressed HGF-stimulated cell proliferation of HepG2 cells (Figs 1c,d and 4f,g) and hepatocytes isolated from adult mice (Fig. 5b), respectively. These observations suggest that Arf6 and PIP5K1A are both dispensable for HGF-regulated cell proliferation of fetal hepatocytes, while HGF-dependent cell proliferation of adult hepatocytes absolutely requires the Arf6-PIP5K1A axis for liver regeneration. It is of interest to clarify a downstream effector of Arf6 functioning in the fetal liver development; its clarification would provide insight into the molecular mechanism of liver development.

HGF is a critical growth factor regulating liver regeneration after liver injuries as well as liver development. Here, we defined a novel HGF signaling pathway regulating adult hepatocyte proliferation that is an essential cell phenomena for the recovery from liver damage. Our findings could provide insight into molecular mechanisms of liver regeneration and into novel therapeutic strategies for hepatic injury caused by infections, toxic materials and surgical resection. In addition, it is of interest to investigate the involvement of c-Met-Arf6-PIP5K1A signaling in hepatocellular carcinoma, since dysregulation of HGF/c-Met is highly related to this disease^7^.

## Materials and Methods

### Mice

Generation of *Pip5k1a* ^−*/−*^ mice with C57BL/6 background is described previously^19, 25^. Female mice of 8–10 weeks old were used for experiments. All experiments with mice were conducted according to the Guidelines for Proper Conduct of Animal Experiments, Science Council of Japan, and protocols were approved by the Animal Care and Use Committee, University of Tsukuba.

### Antibodies and plasmids

All antibodies used in this study are described in Supplementary Table unless otherwise noted. Plasmids expressing C-terminally Flag-tagged wild type (WT) of Arf6 and its constitutively active and dominant negative mutant, Q67L and T44N respectively, were described previously^26^.

### Cell culture, plasmid transfection and HGF treatment of the cell

HepG2 cells were maintained in DMEM supplemented with 10% FBS and 1% antibiotic solution (10,000 units/ml of penicillin and 10 mg/ml of streptomycin) in an atmosphere of 5% CO_2_ at 37 °C. They were transfected with plasmid DNAs or siRNAs using Lipofectamine 2000 (Invitrogen) according to the manufacturer’s instructions. After 48 h incubation, the cells were serum-starved for 12 h, and treated with 10 ng/mL of HGF for the indicated times.

### Assay for measuring the cell number

The number of viable cells was assessed by the trypan blue exclusion assay. Cells were seeded in 12 well plates (5 × 10^4^ cells/well) and transfected with siRNA. After 48 h incubation, cells were treated with HGF for the indicated time, trypsinized and stained with trypan blue. The number of viable cells was counted with a hemocytometer. Five counts were performed per well for three independent experiments.

### Isolation and culture of primary hepatocytes

Hepatocytes of WT and *Pip5k1a* ^−*/−*^ mice (10 weeks old) were isolated as described previously^27^. Isolated hepatocytes showed >80% cell viability assessed by the trypan blue exclusion assay. Cells were plated on collagen-coated plates, cultured and manipulated as described above for HepG2 cells.

### Immunoprecipitation and Western blotting

Cells were lysed in lysis buffer (25 mM Tris-HCl, pH7.5, 1% Triton X-100, 10 mM NaCl, 1 mM EGTA and 5 mM MgCl_2_) containing protease inhibitor cocktail (Nacalai) at 4 °C for 30 min, and centrifuged at 10,000 × g for 10 min. The cell lysates were incubated with the indicated antibodies and protein G/protein A sepharose beads (GE healthcare) or Anti-Flag affinity gels (SIGMA) at 4 °C for 4 h. Immune complexes captured on beads or gels were washed with lysis buffer and eluted with the SDS-PAGE sample buffer by boiling. Eluted proteins were separated by SDS-PAGE, transferred onto a PVDF membrane, and then detected by specific primary antibodies and horseradish peroxidase-conjugated secondary antibodies. Images were quantified using the Image J software (http://rsb.info.nih.gov/ij/).

### Analysis for Arf6 activation

Activation of Arf6 was assessed by the Arf6-GTP pulldown assay as described in the previous report by Santy *et al*.^28^. Briefly, HepG2 cells were seeded on 3.5 cm dishes at 3 × 10^5^ cells/dish and incubated overnight. After 12 h starvation, cells were stimulated with 10 ng/mL of HGF for 10 min. Cells were harvested in lysis buffer (50 mM Tris-HCl, pH 7.5, 100 mM NaCl, 2 mM MgCl_2_, 0.1% sodium dodecyl sulfate, 0.5% sodium deoxycholate, 1% Triton X-100, 10% glycerol, 1 μg/ml of aprotinin, and 1 μg/ml of leupeptin), and lysed at 4 °C for 30 min. The cell extracts were mixed with glutathione *S*-transferase (GST)-GGA3-conjugated glutathione-Sepharose beads and incubated for 30 min with gentle rotation. The beads were washed three times with the washing buffer (50 mM Tris-HCl, pH 7.5, 100 mM NaCl, 2 mM MgCl_2_, 1% NP-40, 10% glycerol, 1 μg/ml of aprotinin, and 1 μg/ml of leupeptin). The Arf6-GTP bound to GST-GGA3 beads was eluted by SDS sample buffer, and detected by Western blotting.

### Lipids extraction and quantification of PIP_3_

Cell Lipids were extracted by the method reported by Grey *et al*.^29^. Cells at 5 × 10^6^ cells/15 cm dish were transfected with siRNAs and treated with HGF as described above. Cells were incubated with ice-cold 0.5 M TCA for 5 min, and centrifuged at 10,000 × g for 5 min. The pellet was resuspended in 5% TCA/1 mM EDTA and centrifuged, then the supernatant was removed. Neutral lipids were extracted from the pellet with MeOH: chloroform (2:1), and the supernatant was discarded. Acidic lipids were then extracted from the pellet with MeOH: chloroform: 12 M HCl (80:40:1). After centrifugation, chloroform and 0.1 M HCl were added to the supernatant and vortexed, followed by centrifugation to separate the organic and aqueous phases. The organic phase was collected and dried in a vacuum dryer. To detect PIP_3_ levels, the extracted lipids were dissolved in 20% DMSO, and spotted on nitrocellulose membranes. The dot membranes were blocked in TBS/0.05% Tween-20 with 5% BSA for 1 h at room temperature and further incubated with anti-PIP_3_ antibody (1:50 dilution; MBL international) for 1 h at room temperature, followed by incubation with horseradish peroxidase*-*conjugated secondary antibodies. Chemiluminescence reagents (Nacalai and Thermo Scientific) were used for detection, and the level of PIP_3_ was quantified by the Image J software.

### Immunocytochemistry

HepG2 cells or primary hepatocytes at 5 × 10^4^ cells/12/dish were seeded on gelatin-coated coverslips in the dish and transfected with siRNAs. After incubation at 37 °C for 48 h, cells were stimulated with HGF as described above, fixed with 4% paraformaldehyde and permeabilized with 0.5% Triton-X100. Cells were then blocked with 1% BSA in PBS and immunostained with indicated primary antibodies. The fluorescent-labeled secondary antibodies and DAPI were used to detect target proteins and the nucleus, respectively.

### Partial hepatectomy

Eight weeks old female mice were used for partial hepatectomy (PH). Mice were anesthetized with isoflurane and subjected to 70% liver resection of the median and left lateral lobes as previously described^30^. The mice were euthanized at days after PH described in the figure. Livers were isolated from the mice, and liver weight/body weight was determined. A part of the liver tissue was fixed in 4% paraformaldehyde/PBS and then embedded in O.C.T. (Tissue-Tek®). For measuring serum aspartate aminotransferase (AST) and alanine transaminase (ALT) activities, blood was collected from the submandibular vein before sacrifice. AST and ALT activities were measured with the Amplite^TM^ fluorimetric assay kit (AAT Bioquest, Inc.).

To measure hepatocyte proliferative activity, the livers removed from mice in the indicated times (0, 2 and 5 days) were fixed in 4% paraformaldehyde/PBS at 4 °C overnight, embedded in O.C.T., and sliced into 10 μm sections. Sections were permeabilized and blocked with 0.1% Triton-X/5% BSA/PBS for 30 min at room temperature, and then stained with anti-Ki-67 antibody (Abcam, 1:150 in 5% BSA/PBS) and anti-albumin antibody (Bethyl, 1:150 in 5% BSA/PBS) at 4 °C overnight. After washing with PBS, sections were stained with secondary antibodies and DAPI. The Ki-67-positive cells were counted and analyzed using the BZ-II Analyzer (KEYENCE).

### Statistical analysis

All quantified data were expressed as means ± SEM and analyzed by Student *t*-test, one-way ANOVA with post hoc Tukey’s test, or two-way ANOVA with post hoc Bonferroni’s test using the Graphpad Prism 5 software.

## Electronic supplementary material

Supplementary Information
