# Supplementary material for: Regulation of HGF-induced hepatocyte proliferation by the small GTPase Arf6 through the PIP2-producing enzyme PIP5K1A
Source: Sci Rep. 2017 Aug 25;7:9438. doi: 10.1038/s41598-017-09633-z (PMC5572707; doi:10.1038/s41598-017-09633-z)
Supplement: Supplementary file 1 — Supplementary Information [file 41598_2017_9633_MOESM1_ESM.pdf]

Supplementary Information

Regulation of HGF-induced hepatocyte proliferation by the small GTPase Arf6 through the PIP_2-producing enzyme PIP5K1A

Meng-Tsz Tsai, Naohiro Katagiri, Norihiko Ohbayashi, Kenichi Iwasaki, Nobuhiro Ohkohchi, Shih-Torng Ding, Yasunori Kanaho, and Yuji Funakoshi

Table of Contents

Supplementary Table……………………………….2

Supplementary Figure……………………………...4

Supplementary Reference………………………......6

Supplementary Table. List of antibodies.

**Antibody**	**Catalog No.**	**Company**
Rabbit anti-AKT	9272S	Cell Signaling Technologies
Rabbit anti-phospho-AKT (Ser473)	9271	
Rabbit anti-phospho-Erk1/2 (Thr202/Tyr204)	9101S	
Rabbit anti-Erk1/2	9102	
Rabbit anti-phospho-c-Met	3126	
Rabbit anti-Actin	A2066	SIGMA
Mouse anti-α-tubulin	T6199	
Mouse anti-Flag	F3165	
Rabbit anti-Ki-67	ab15580	Abcam
Rabbit anti-c-Met (C-12)	sc-10	Santa Cruz
Goat anti-PIP5K1A	sc-11774	
Goat anti-PIP5K1C	sc-11786	
Goat anti-Albumin	A90-134P	Bethyl
Mouse anti-PIP_3 IgG	D145-3	MBL
Mouse anti-PIP_2 IgM	Z-P045	Echelon Biosciences
Rabbit anti-Arf6	Self-prepared antibodies [1]	
Rabbit anti-PIP5K1A		
Rabbit anti-PIP5K1B		
Horseradish peroxidase-conjugated anti-rabbit secondary antibody	7074S	Cell Signaling Technologies
Horseradish peroxidase-conjugated anti-mouse secondary antibody	7076S	

Alexa488-conjugated anti-rabbit IgG	A11034	Invitrogen
Alexa488-conjugated anti-mouse IgG	A11029	
Alexa647- conjugated anti-goat IgG	A21447	
Cy3-conjugated anti-mouse IgM	115-165-075	Jackson ImmunoResearch

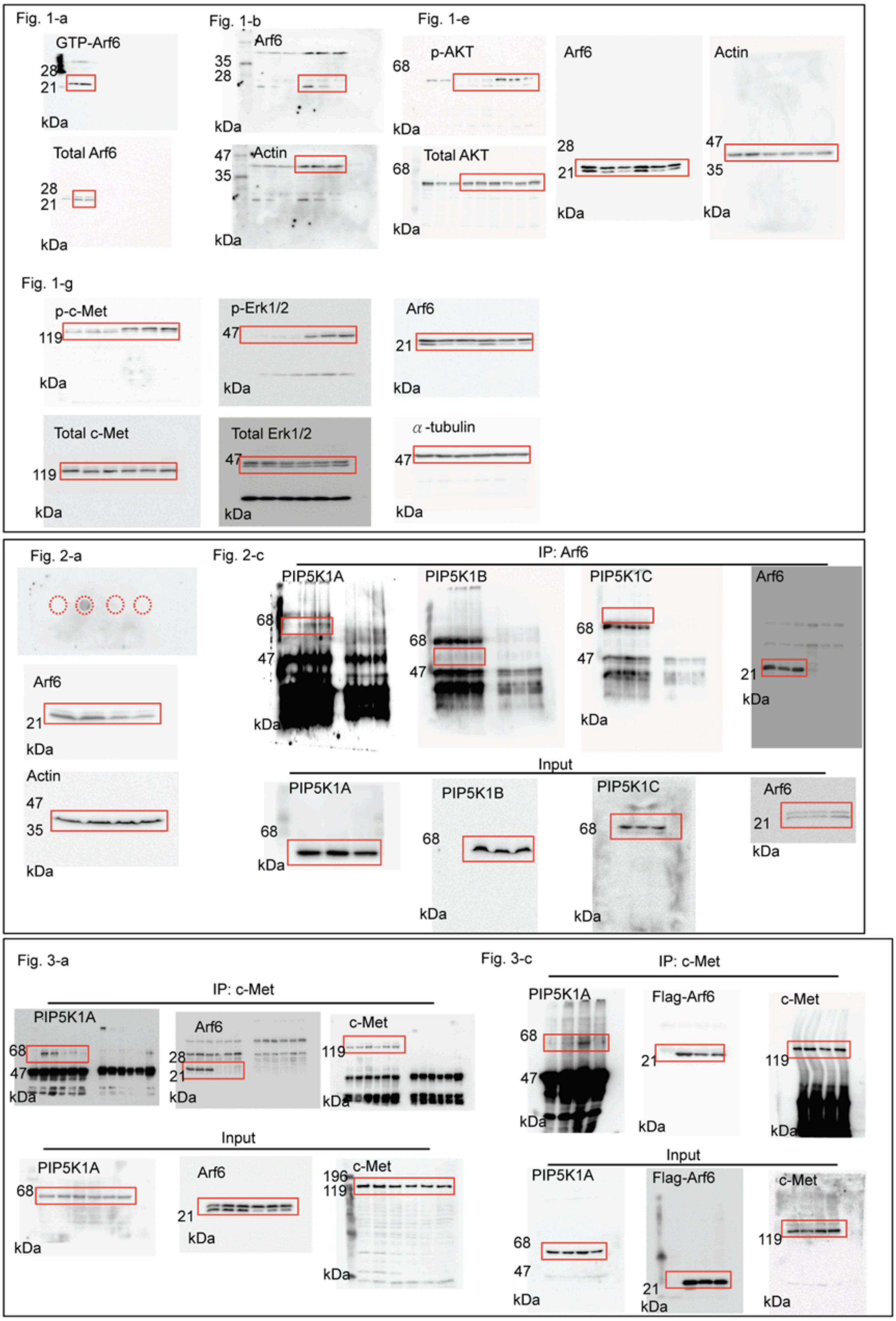

Supplementary Figure. Uncropped images of blots.

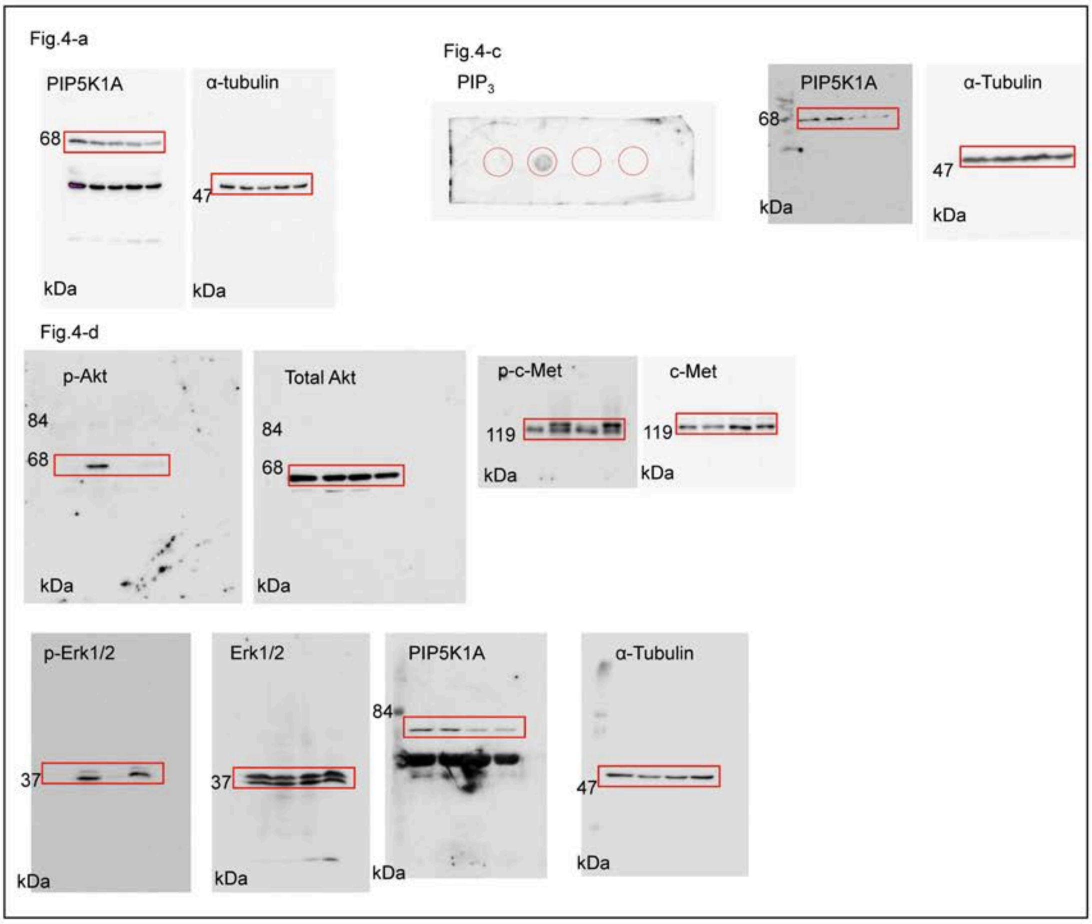

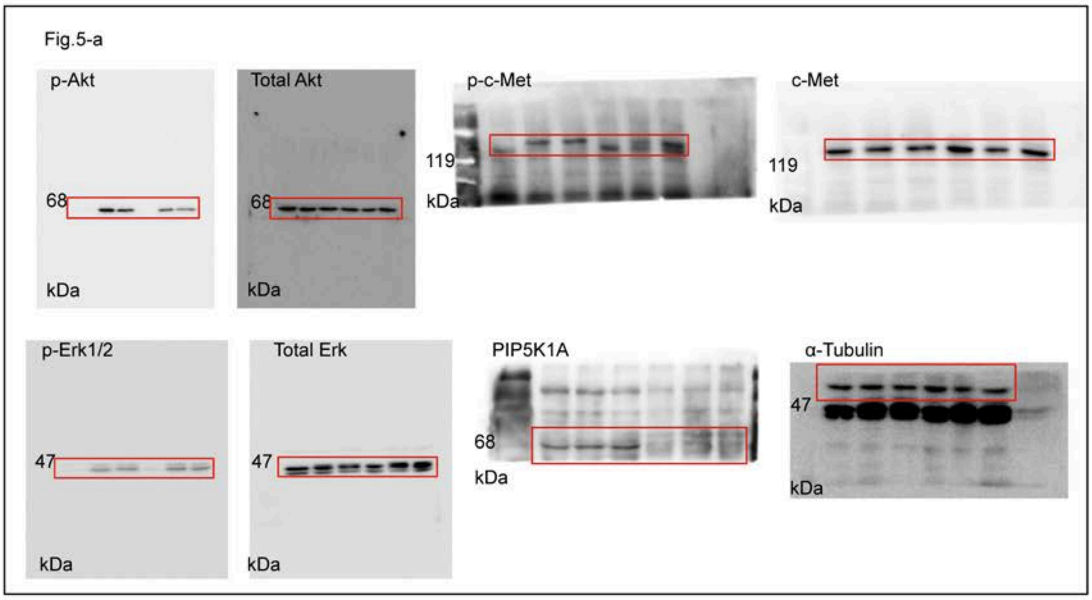

Supplementary Figure. Uncropped images of blots.

Supplementary Reference

1. Hasegawa H, Noguchi J, Yamashita M, Okada R, Sugimoto R, Furuya M, et al. Phosphatidylinositol 4-phosphate 5-kinase is indispensable for mouse spermatogenesis. *Biol Reprod* **86**, 136, 131-112 (2012).
